# Research Advances in Targeted Modulation of Macrophage M1/M2 Polarization for the Treatment of Rheumatoid Arthritis

**DOI:** 10.1155/jimr/4203230

**Published:** 2026-11-02

**Authors:** Hui Zuo, Enqi Fan, Long Zeng, Jun Hu, Jiajia Sang, Feng Hao

**Affiliations:** ^1^ Nanjing University of Chinese Medicine Nanjing 210023 China njucm.edu.cn; ^2^ The Affiliated Hospital of Nanjing University of Chinese Medicine Nanjing 210029 China njucm.edu.cn

**Keywords:** M1/M2 polarization, macrophage, metabolic reprogramming, rheumatoid arthritis, signaling pathways, targeted therapy, traditional Chinese medicine

## Abstract

Rheumatoid arthritis (RA) is a disease of the immune system that leads to continuous synovial inflammation and the progressive breakdown of joints. As essential effector cells in innate immunity, macrophages can become either pro‐inflammatory M1 or anti‐inflammatory M2 phenotypes, playing a critical role in RA pathogenesis. A marked M1/M2 polarization imbalance exists in the synovial microenvironment of RA, in which excessive activation and accumulation of M1 macrophages serve as a key hub driving persistent inflammation, cartilage degradation, and bone erosion. Currently, conventional disease‐modifying antirheumatic drugs (DMARDs) with methotrexate (MTX) as the anchor agent remain the mainstay for RA management; however, approximately 30%‐40% of RA patients show an inadequate response to MTX, resulting in persistent synovial inflammation, progressive joint damage, and difficult‐to‐control disease. For macrophage‐directed phenotypic modulation in RA therapy, novel smart nanodelivery systems are a key research focus, while accumulating preclinical evidence supports the capacity of traditional Chinese medicine (TCM) to restore M1/M2 homeostasis. This review systematically summarizes the polarization mechanisms of macrophages in RA, their core pathological functions, and recent advances in targeting these cells as therapeutic strategies, therefore delivering novel insights and references for RA treatment through targeted changes in macrophage polarization.

## 1. Introduction

Rheumatoid arthritis (RA) is an autoimmune disorder that mainly causes symmetric and erosive inflammation in the joints, primarily impacting the small joints in the extremities, with a worldwide prevalence of about 0.46% [1]. The primary pathological feature of RA is synovitis, which, as the disease advances, leads to permanent damage to cartilage and bone, eventually causing joint deformity and loss of function [2]. cDMARDs, with methotrexate (MTX) as the core anchor agent, constitute the primary therapeutic backbone for RA. These agents confer potent effects on suppressing disease activity and slowing joint destruction. Nonetheless, 30%–40% of RA patients manifest an inadequate response to MTX, which causes persistent synovitis, progressive joint injury, and refractory disease status [3]. RA’s pathogenesis is complicated, and recent findings point to macrophage polarization as a crucial element.

Adjusting the macrophage phenotype and function in response to microenvironmental signals is known as macrophage polarization. The direction of polarization is governed by transcription factors, epigenetic mechanisms, and the tissue microenvironment [4], and M2 macrophages are also referred to as alternatively activated. M1 macrophages mainly produce pro‐inflammatory cytokines, including tumor necrosis factor‐α (TNF‐α), interleukin‐1β (IL‐1β), and IL‐6, thereby driving inflammation and tissue destruction; M2 macrophages mainly produce anti‐inflammatory cytokines, such as IL‐10 and transforming growth factor‐β (TGF‐β), thereby promoting tissue repair and maintaining homeostasis [5]. In RA patients, M1 macrophages are markedly increased in the synovium and circulation, whereas M2 macrophages are relatively deficient [6]. The M1/M2 ratio is positively correlated with disease activity and the severity of bone erosion [7]. Thus, addressing the imbalance in macrophage polarization has become a vital research strategy for reestablishing immune homeostasis and achieving targeted therapy in RA. This review comprehensively outlines the polarization mechanisms of M1 and M2 macrophages in RA and their involvement in disease development, while also discussing recent progress in targeted treatments. This review seeks to deliver novel insights and references for the focused regulation of macrophage polarization in RA therapy.

Literature search strategy: A structured literature search was conducted in PubMed and the Web of Science for publications from June 2021 to June 2026. The search terms comprised combinations of the following keywords: RA, macrophage, M1/M2 polarization, signaling pathways, metabolic reprogramming, targeted therapy, and traditional Chinese medicine (TCM). We prioritized original research articles and relevant reviews that addressed macrophage polarization mechanisms, macrophage heterogeneity, regulatory mechanisms of polarization, targeted therapeutic strategies, and TCM‐mediated modulation in RA. Studies unrelated to RA or without direct relevance to macrophage polarization were excluded. Studies derived from other disease contexts were considered only when they provided mechanistic insights into macrophage polarization; such evidence was treated as supportive rather than RA‐specific. Additionally, the reference lists of key publications were screened to identify further relevant records. This is a narrative review, not a systematic review; accordingly, study selection was not performed in accordance with a formal systematic‐review protocol.

## 2. Macrophage Origin and Heterogeneity

Macrophages, which play a crucial role in the body’s innate immune system, were first identified in starfish larvae in 1882 by the Russian‐born naturalist Ilya (Élie) Metchnikoff [8]. Based on their origin, macrophages can generally be divided into tissue‐resident macrophages (TRMs) and monocyte‐derived macrophages (MDMs). Yolk‐sac‐derived and fetal‐liver‐derived progenitors give rise to TRMs, whereas monocytes generate MDMs. TRMs possess self‐renewal capacity and maintain tissue homeostasis. By contrast, MDMs are generated under pathological conditions [9, 10]. In RA, the relationship between the origin and function of synovial macrophages remains poorly defined, and it is still uncertain where specific myeloid cell subsets in the human synovium originate from. Therefore, elucidating the precise origin of synovial macrophages in RA patients will provide an opportunity to develop precision interventions that target the origin of particular cell subsets [11]. Studies have shown that TRMs, which are seeded during embryonic development, and MDMs, which are recruited from peripheral blood monocytes under inflammatory conditions, coexist within RA joints [12], with the latter having a major impact on inflammation in the joints. Single‐cell sequencing technology has further revealed the complex lineage composition of RA synovial macrophages, including a pro‐inflammatory subset characterized by CD48^high^S100A12^+^ cells that secrete pro‐inflammatory cytokines; anti‐inflammatory and reparative subsets, such as TREM2^high^, FOLR2^high^LYVE1^+^ macrophages; and functionally defined subsets such as CD48^+^SPP1^+^ cells. Collectively, these subsets constitute a continuous phenotypic spectrum that transcends the conventional M1/M2 dichotomy [9]. Such phenotypic diversity is intimately linked to distinct metabolic features: glycolysis is the primary energy source for M1 macrophages, whereas M2 macrophages depend on oxidative phosphorylation (OXPHOS) [13]. Different subsets also exhibit differential responses to the same microenvironmental signals and varying susceptibility to apoptosis or ferroptosis; for instance, M2 macrophages are more prone to ferroptosis under iron overload conditions [14]. Therefore, recognizing macrophage heterogeneity is crucial for developing novel RA therapies that precisely target pathogenic subsets while preserving immune homeostasis.

Although recent single‐cell studies indicate that macrophage activation states are more diverse than the conventional M1/M2 classification, the M1/M2 framework still serves as a practical reference for defining functional extremes in RA pathogenesis and therapeutic regulation. This review adopts the M1/M2 nomenclature while acknowledging its inherent limitations.

## 3. Macrophage Polarization and Its Regulatory Mechanisms in RA

### 3.1. M1 Macrophage Polarization in RA

The excessive activation of M1 macrophages is crucial in the development of RA, and they predominantly infiltrate the sublining layer of the synovium. There, the infiltrated M1 macrophages act as key effector cells, promoting continuous synovial inflammation and joint destruction via the secretion of inflammatory cytokines. Furthermore, circulating peripheral blood monocytes, which are closely related to the origin of synovial tissue macrophages, also exhibit sustained hyperinflammatory and metabolically activated M1 phenotypic characteristics in RA patients [15]. Studies have confirmed a marked M1/M2 polarization imbalance in synovial macrophages from RA patients, characterized by a significant increase in CD86^+^ cells and a decrease in CD206^+^ cells [16]. M1 macrophages are responsible for the production and secretion of pro‐inflammatory cytokines, including IL‐1β, IL‐6, and TNF‐α, which directly encourage the growth and activation of fibroblast‐like synoviocytes (FLS) and prompt them to release more inflammatory mediators and matrix metalloproteinases (MMPs), thereby initiating and exacerbating synovial inflammation. Furthermore, M1 macrophages exhibit high expression of major histocompatibility complex class II (MHC II) molecules and co‐stimulatory molecules (e.g., CD80 and CD86), thereby conferring potent antigen‐presenting capacity. This enables them to activate naive T cells, especially Th1 and Th17 cells, thereby initiating and amplifying T cell‐dependent adaptive immune responses and establishing a broader inflammatory network [17]. Simultaneously, this mechanism promotes the activation, multiplication, and transformation of B cells into plasma cells, which subsequently generate autoantibodies like rheumatoid factor and anti‐citrullinated protein antibodies [18]. The immune complexes formed by these autoantibodies can activate macrophages via Fc receptors and the complement system, driving them toward an M1 phenotype [19]. Furthermore, immune complexes created by anti‐citrullinated protein antibodies and citrullinated proteins can engage with macrophages via toll‐like receptor 4 (TLR4) and Fc gamma receptor II (FcγRII), boosting the creation of pro‐inflammatory elements such as TNF‐α [20]. In RA, activated macrophages release chemokines that consistently attract peripheral blood monocytes to the synovium; these newly recruited monocytes further differentiate into M1 macrophages within the local pro‐inflammatory microenvironment, perpetuating and amplifying the inflammation [21]. Additionally, M1 macrophage polarization constitutes the core pathological process driving joint damage and bone erosion. These cells significantly increase receptor activator of nuclear factor‐κB ligand (RANKL) expression and decrease osteoprotegerin (OPG) levels, which encourages osteoclast differentiation and bone resorption [22]. They also emit large amounts of pro‐inflammatory substances such as IL‐1β, TNF‐α, and IL‐6, along with chemokines and MMPs, which worsen synovial inflammation, encourage pannus development, and result in the damage of nearby cartilage and bone tissue [18] (Figure 1).

**Figure 1:**
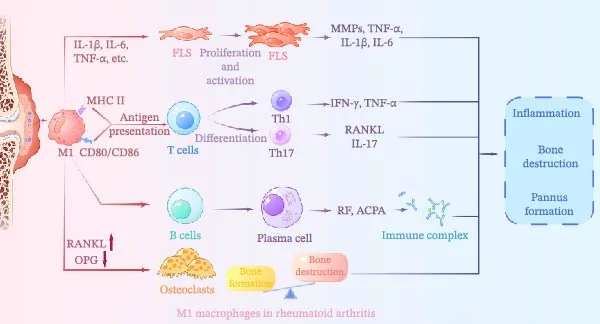
M1 macrophages in rheumatoid arthritis. In RA, M1 macrophages are pivotal in the production and release of pro‐inflammatory cytokines, including IL‐1β, IL‐6, and TNF‐α. These cytokines exert a direct stimulatory effect on FLS, which subsequently secrete additional pro‐inflammatory mediators, thereby exacerbating the inflammatory response. Furthermore, M1 macrophages exhibit high expression levels of MHC II molecules and co‐stimulatory molecules, such as CD80 and CD86, endowing them with a potent antigen‐presenting capacity that activates Th1 and Th17 cells. This activation initiates and propagates T cell‐dependent adaptive immune responses, thereby establishing an extensive inflammatory network. Additionally, M1 macrophages facilitate B cell proliferation and differentiation into plasma cells that produce autoantibodies, with the resultant immune complexes further intensifying inflammation at the lesion site. Moreover, M1 macrophages upregulate RANKL and downregulate OPG, thereby promoting osteoclast differentiation and bone resorption, which exacerbates inflammation, bone erosion, and pannus formation.

### 3.2. M2 Macrophage Polarization in RA

M2 macrophages exert protective effects in RA by suppressing inflammation, promoting tissue repair, and alleviating bone destruction, thereby ameliorating the disease [23]. M2 macrophages produce a range of anti‐inflammatory cytokines, including IL‐10, IL‐4, TGF‐β, vascular endothelial growth factor (VEGF), and platelet‐derived growth factor (PDGF). These cytokines suppress T‐cell activation and growth, are involved in Th2‐type immune responses, and aid in tissue repair and the formation of new blood vessels [8, 24]. In RA, M2 macrophages play a crucial role in controlling the differentiation of osteogenic cells and the formation of the bone. The anti‐inflammatory substances they release can prevent osteoclast differentiation and activation by lowering the expression of the RANK and tartrate‐resistant acid phosphatase (TRAP) enzyme associated with osteoclasts [25]. Transforming macrophages from the M1 to the M2 phenotype can boost osteoblast growth, attachment, and mineralization, while also increasing the expression of genes related to bone formation, including runt‐related transcription factor 2 (RUNX2), alkaline phosphatase (ALP), collagen type I alpha 1 chain (COL1A1), osteopontin (OPN), and osteocalcin (OCN) [26] (Figure 2).

**Figure 2:**
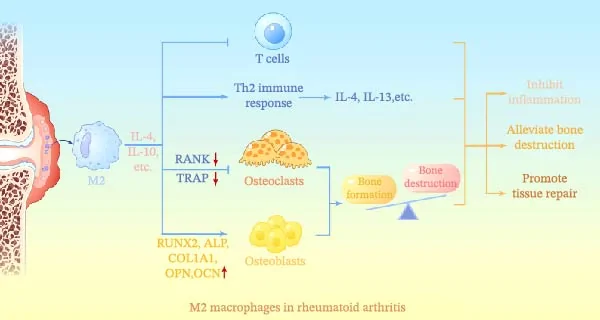
M2 macrophages in rheumatoid arthritis. In RA, M2 macrophages are characterized by their secretion of anti‐inflammatory cytokines, including IL‐10 and IL‐4. These cytokines play a pivotal role in inhibiting T cell activation and proliferation, facilitating Th2‐type immune responses, and inducing the production of additional anti‐inflammatory cytokines such as IL‐4 and IL‐13. Furthermore, M2 macrophages impede osteoclast differentiation and activation through the downregulation of RANK and TRAP expression. In addition, M2‐polarized macrophages enhance the expression of osteogenic genes, including RUNX2, ALP, COL1A1, OPN, and OCN. Collectively, these activities of M2 macrophages mitigate the inflammation and bone destruction mediated by M1 macrophages, thereby contributing to the restoration of joint homeostasis.

A popular research focus in RA treatment is on altering macrophage polarization to the M2 phenotype or converting M1 macrophages into the M2 phenotype. Enhancing M2 macrophage function can considerably ease RA progression. Research [27] discovered that altering the miR‐33/NLRP3 pathway can lead to macrophages polarizing into an M2 phenotype. Cell‐based therapies have demonstrated the protective effects of M2 macrophages; for example, exosomes derived from adipose stem cells (ADSCs‐EXO‐ICA) have been shown to effectively inhibit glycolysis by targeting the ERK1/2/HIF‐1α/GLUT1 pathway, therefore blocking the shift of macrophages to the M1 phenotype and supporting their polarization to M2, thus alleviating inflammation and protecting cartilage [28]. Moreover, the CTLA4‐Ig fusion protein abatacept has demonstrated the ability to transform monocytes/macrophages from RA patients from the M1 to the M2 phenotype in vitro [29]. Therefore, the protective effects of M2 macrophages are reflected not only in their inherent anti‐inflammatory and tissue‐repair functions, but also in the remarkable plasticity of macrophages that enables their conversion from a pro‐inflammatory state to a protective phenotype. This transition represents one of the core therapeutic strategies for restoring immune homeostasis in the joint and achieving disease remission.

### 3.3. Driving Factors of Macrophage Polarization Imbalance in RA

#### 3.3.1. Aberrant Activation of Signaling Pathways

Several signaling pathways are essential in managing the imbalance between M1/M2 polarization. Previous research has confirmed that this process is accurately controlled by pathways like nuclear factor‐kappa B (NF‐κB), Janus kinase/signal transducer and activator of transcription (JAK/STAT), mitogen‐activated protein kinase (MAPK), Notch, and phosphatidylinositol 3‐kinase/protein kinase B (PI3K/Akt). Figures 3 and 4 display the regulatory mechanisms of each pathway in macrophage polarization, which are elaborated later in the study.

**Figure 3:**
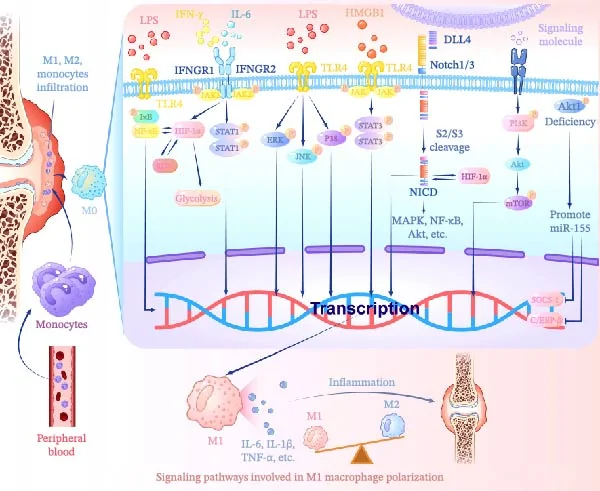
Signaling pathways involved in M1 macrophage polarization. In RA, circulating monocytes migrate to the inflamed synovium and differentiate into M0 macrophages, which polarize to the M1 phenotype due to various intracellular signaling pathways, resulting in an M1/M2 imbalance. This polarization leads to the release of pro‐inflammatory cytokines like IL‐6, IL‐1β, and TNF‐α, worsening synovial inflammation. (1) NF‐κB pathway: LPS binds to TLR4, activating the NF‐κB pathway. NF‐κB and HIF‐1α mutually enhance each other, and in the hypoxic RA environment, HIF‐1α drives glycolysis and ROS accumulation. This ROS stabilizes HIF‐1α, creating a feedback loop that promotes M1 polarization and increases joint inflammation and damage. (2) JAK/STAT pathway: IFN‐γ and IL‐6 activate the JAK1/JAK2‐STAT1 pathway, increasing HIF‐1α expression. Additionally, HMGB1 can activate the JAK/STAT3 pathway via TLR4, further promoting M1 polarization. (3) MAPK pathway: LPS activates TLR4, leading to p38, ERK, and JNK activation, which promotes M1 polarization. (4) Notch pathway: DLL4 binding to Notch1/3 receptors on macrophages triggers cleavage events that release notch intracellular domain, which enters the nucleus and activates NF‐κB, MAPK, and Akt pathways, enhancing M1 marker expression. (5) PI3K/Akt pathway: ablation of Akt1 elevates miR‐155 levels, repressing its target genes SOCS‐1 and C/EBP‐β to favor the M1 macrophage phenotype. M0, unpolarized resting macrophages.

**Figure 4:**
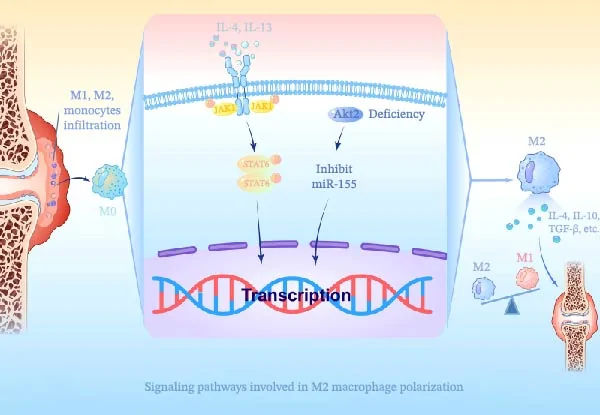
Signaling pathways involved in M2 macrophage polarization. In RA, IL‐4 and IL‐13 bind to receptors on M0 macrophages. This activates JAK1, which in turn phosphorylates STAT6. Phosphorylated STAT6 forms dimers and enters the nucleus. This process promotes M2 polarization. Deletion of AKT2 suppresses miR‐155 expression and skews macrophages toward the M2 phenotype. M2 macrophages then release anti‐inflammatory cytokines, such as IL‐4, IL‐10, and TGF‐β. These cytokines correct the M1/M2 imbalance and ameliorate synovial inflammation. Tissue repair is subsequently promoted. M0, unpolarized resting macrophages.

##### 3.3.1.1. NF‐κB

In the progression of RA, NF‐κB is an essential transcription factor that stimulates M1 macrophage activation. In RA, stimuli, such as lipopolysaccharide (LPS), TNF‐α, and IL‐1β, can activate the NF‐κB pathway through receptors such as TLR4 on the macrophage surface, promoting the release of M1 pro‐inflammatory cytokines, like IL‐1β, IL‐6, and TNF‐α, thus creating a self‐perpetuating inflammatory cycle [30]. Moreover, this pathway reciprocally upregulates hypoxia‐inducible factor 1α (HIF‐1α) within the synovial microenvironment [31], jointly activating the NF‐κB/HIF‐1α axis, which causes an increase in reactive oxygen species (ROS) and promotes M1 macrophage polarization, accelerating disease progression. Thus, controlling the imbalance of macrophage polarization through the NF‐κB signaling pathway aids in reducing inflammatory infiltration in the synovial tissue of RA, offering a possible intervention strategy for managing RA clinically.

##### 3.3.1.2. JAK/STAT

In RA, the JAK/STAT pathway’s abnormal activation usually encourages M1 macrophage polarization and suppresses M2 polarization, thus continuing the harmful inflammation cycle. The JAK protein family comprises four receptor‐associated tyrosine kinases—JAK1, JAK2, JAK3, and TYK2—while the STAT family encompasses signal transducer and activator of transcription proteins, including STAT1, STAT2, STAT3, STAT4, STAT5a/b, and STAT6. Among them, IFN‐γ and IL‐6 activate STAT1 via JAK1/JAK2, upregulate HIF‐1α, and promote glycolysis, thus providing energy for M1 macrophages to amplify inflammation [32, 33]. Recent studies have further revealed that chemokines, such as CCL7, can form an autocrine positive‐feedback loop via CCR1/JAK2/STAT1, thereby enhancing M1 polarization [34]. On the other hand, IL‐4/IL‐13 can induce phosphorylation of STAT6, resulting in M2 polarization, decreased inflammation, and improved tissue repair [35]. Notably, the regulatory role of STAT3 is microenvironment‐dependent: in the iron‐enriched milieu of the RA synovium, M2 macrophages are more prone to ferroptosis, resulting in an M1/M2 imbalance; high‐mobility group box‐1 (HMGB1) released from these ferroptotic M2 binds to TLR4 on M1 macrophages, triggering site‐specific phosphorylation of STAT3 at Ser727, which boosts the production of pro‐inflammatory elements such as IL‐6 in M1 macrophages, further exacerbating inflammation [36]. Paradoxically, in the tumor microenvironment, deoxycholic acid can activate the TGR5/STAT3 signaling pathway via TGR5 receptor activation, thereby promoting M2 macrophage polarization [37]. An in‐depth investigation of STAT3’s differential responses across distinct microenvironments may yield novel strategies for the targeted modulation of the M1/M2 balance.

##### 3.3.1.3. MAPK

The MAPK family primarily includes ERK1/2, JNK1/2/3, and p38, which convert signals from outside the cell into responses within the cell and are crucial in managing the M1/M2 polarization equilibrium in RA. Through TLR4, LPS triggers p38/JNK and starts the NF‐κB pathway, leading to M1 polarization and inflammation. Thus, inhibiting p38/JNK phosphorylation can attenuate M1 polarization [38]. The pro‐inflammatory effects of the MAPK pathway are intricately linked to other signaling cascades. Research indicates that Nesfatin‐1 enhances CCL2 expression dependent on NF‐κB through the MEK/ERK and p38 pathways, facilitating monocyte movement and encouraging M1 macrophage polarization [39]. In RA joints, elevated CCL25/CCR9 signaling promotes monocyte migration and polarization toward an atypical M1 phenotype via p38/ERK pathway activation, leading to IL‐8 and CCL2 secretion while inhibiting phagocytic function [40]. By blocking the CCL25/CCR9 axis and inhibiting the MAPK pathway, the differentiation of pro‐inflammatory macrophages can be decreased, suggesting a new strategy for targeted regulation of macrophage polarization in the treatment of RA. In addition, neuroimmunological studies have demonstrated that nerve growth factor (NGF) binds to the TrkA receptor to activate the MEK/ERK pathway, promote the phosphorylation of the AP‐1 transcription factor (c‐Jun), and upregulate ICAM‐1 expression in FLS, thereby enhancing monocyte adhesion. Simultaneously, FLS‐derived secretions induce M1 macrophage polarization and intensify inflammation in RA. Targeting the NGF/TrkA/MEK/ERK axis is expected to suppress M1 macrophage polarization and monocyte infiltration, thereby alleviating RA progression [41].

##### 3.3.1.4. Notch

In the RA synovial tissue, the Notch signaling pathway, a highly conserved regulatory mechanism, is often activated abnormally. The stimulation of the Notch pathway leads to M1 polarization and the emission of pro‐inflammatory cytokines, wherein delta‐like ligand 4 (DLL4)‐induced Notch signaling enhances the transcription of pro‐inflammatory genes and increases M1 macrophage numbers by activating the MAPK, NF‐κB, and Akt pathways [42]. Conversely, inhibiting the Notch pathway can encourage macrophages to polarize towards the M2 phenotype, thereby ameliorating RA symptoms and protecting the joint and surrounding tissues [43]. In the RA synovial tissue, Notch‐1/3 and HIF‐1α are highly expressed. Under hypoxic microenvironmental conditions, HIF‐1α directly upregulates Notch‐1/3 transcription. Their downstream effectors N1ICD/N3ICD cooperatively drive FLS activation, thereby exacerbating synovial hyperplasia, inflammation, and joint destruction [44]. Therefore, modulating the Notch pathway to correct the M1/M2 polarization imbalance may alter the inflammatory trajectory of RA and promote synovial tissue repair.

##### 3.3.1.5. PI3K/Akt

The PI3K/Akt pathway plays a key role in the regulation of macrophage polarization, and its overactivation leads to increased M1 polarization and worsens inflammation. PI3K is a phosphatidylinositol kinase found inside cells, possessing serine/threonine kinase activity, and is made up of a heterodimer that functions as a protein kinase through its p110 catalytic subunit and p85 regulatory subunit. Akt, which is also referred to as protein kinase B, is made up of three isoforms: Akt1, Akt2, and Akt3, and it serves as the key effector molecule of PI3K. Studies indicate that the influence of the PI3K/Akt pathway on M1/M2 polarization is isoform‐dependent: genetic ablation of Akt1 elevates miR‐155 expression, which in turn represses its target genes suppressor of cytokine signaling‐1 (SOCS‐1) and CCAAT/enhancer‐binding protein‐β (C/EBP‐β) and drives M1‐type macrophage polarization. In contrast, Akt2 ablation reduces miR‐155 abundance, derepressing SOCS‐1 and C/EBP‐β, and consequently favoring M2 polarization [45, 46]. Moreover, ADP‐ribosylation factor‐like 4C, which is highly expressed in RA FLS, can alter inflammatory cytokine secretion by activating the PI3K/Akt and MAPK pathways, thereby driving macrophage M1 polarization in a paracrine manner [47]. Consequently, given that genetic ablation of Akt2 promotes M2‐type macrophage polarization, pharmacological inhibition of this isoform is expected to recapitulate such phenotypic effects. This may increase the abundance of M2 macrophages within the synovial tissue, facilitate anti‐inflammatory responses and tissue repair, and provide new research perspectives for the precise modulation of M1/M2 polarization in RA therapy.

#### 3.3.2. Metabolic Reprogramming

Previous studies largely regarded macrophage polarization as a passive response to specific cytokines. However, recent research has revealed that a single signal does not drive this process; rather, it is closely linked to macrophages’ metabolic patterns. Under inflammatory stimuli, macrophages undergo metabolic reprogramming, which in turn governs their functional phenotypic switch [48]. This finding has offered a new viewpoint on how macrophages function in RA. Within the pathological microenvironment of RA, M1 and M2 macrophages have significantly different metabolic characteristics. M1 macrophages primarily rely on glycolysis for rapid ATP and lactate production, supporting their pro‐inflammatory activities; in comparison, M2 macrophages depend more on OXPHOS, fatty acid oxidation, and glutamine metabolism to support anti‐inflammatory and tissue repair activities [49]. Due to excessive cell proliferation and vascular dysfunction, the synovium in RA pathology swiftly establishes a localized hypoxic environment, which can upregulate HIF‐1α expression. HIF‐1α activation is, in turn, a critical factor driving aerobic glycolysis and M1 macrophage polarization, while simultaneously increasing the release of pro‐inflammatory cytokines, such as IL‐1β, to reinforce M1 polarization. Therefore, inhibiting HIF‐1α or key glycolytic enzyme activities can effectively attenuate macrophage pro‐inflammatory responses [50, 51]. Notably, there is a bidirectional regulatory relationship between synovial macrophages and the local microenvironment. Macrophages are highly sensitive to changes in metabolite concentrations in the microenvironment, while their own metabolic activities can, in turn, reshape the microenvironment by producing or consuming metabolites. In M1 macrophages, the tricarboxylic acid cycle (TCA), OXPHOS, and fatty acid oxidation are inhibited, causing a deficiency in mitochondrial OXPHOS and leading to the buildup of TCA intermediates like citrate and succinate. Among these, succinate accumulation induces ROS generation, further stabilizes HIF‐1α expression, and simultaneously upregulates glycolysis‐related genes such as solute carrier family 2 member 1 and lactate dehydrogenase A [52]. In contrast, citrate accumulation interferes with the TCA cycle by increasing the mitochondrial citrate carrier and reducing isocitrate dehydrogenase activity. The aberrant accumulation of these two intermediates synergistically drives M1 polarization. Accordingly, targeted clearance of succinate and citrate or inhibition of related pathways can effectively curb excessive glycolytic activation in M1 macrophages. In addition to glycolysis, amino acid metabolism also influences macrophage polarization. Studies have shown that when macrophages utilize arginine via the nitric oxide synthesis pathway, M1 polarization is promoted [53]. Moreover, glutamine metabolism rate also plays a role in macrophage phenotype, where glutamine deficiency can lower the expression of genes specific to M2, such as IRF4, KLF4, and CD206, by modulating H3K27 methylation at gene promoter sites [54]. In the glucose‐scarce synovial microenvironment of RA, CCL18‐triggered RFX5‐GLUD1 signaling enables synovial macrophages to utilize glutamate for energy supply and cope with nutrient deprivation while maintaining robust antigen‐presenting activity [55]. Nevertheless, such RFX5‐mediated metabolic resilience cannot guarantee permanent cell survival. The reparative‐phenotype SELENOP^hi^MerTK^+^CD206^+^ synovial macrophage subset, even equipped with glutamine‐fueled survival machinery, is vulnerable to an autocrine C1q‐C1QBP‐initiated metabolic crisis. C1q‐C1QBP complex translocates to mitochondria and activates NAD^+^ hydrolase SARM1, which triggers massive NAD^+^ consumption, accumulation of second‐messenger cADPR, and ultimately NLRC5/NLRP12‐driven PANoptotic lytic cell death. Contents released from dying macrophages further perpetuate local synovial inflammation [56]. This reveals that glutaminolysis‐supported metabolic fitness and immunogenic PANoptotic cell death represent two opposite metabolic fates for synovial macrophages under RA pathological conditions. Moreover, the overproduction of Indoleamine 2,3‐dioxygenase (IDO), which is the key enzyme in the tryptophan–kynurenine pathway, can lead to the polarization of M2 macrophages. In contrast, IDO gene knockout confers a pro‐inflammatory phenotype [57], suggesting that dysregulation of amino acid metabolism may be an important contributor to abnormal macrophage polarization in RA. Finally, abnormal lipid metabolism plays a role in controlling macrophage polarization. In the context of RA, disturbances in lipid metabolism can lead to significant ROS production and trigger pro‐inflammatory pathways such as NF‐κB, and suppress glutathione peroxidase 4 (GPx4) activity, rendering M2 macrophages—which are inherently sensitive to ferroptosis—even more susceptible to ferroptosis; the HMGB1 released from ferroptotic M2 macrophages activates M1 macrophages through the TLR4/STAT3 axis, exacerbating the M1/M2 imbalance and thereby aggravating RA inflammation and tissue damage [36, 58]. In summary, the various metabolic pathways in macrophages in RA are interconnected and mutually influential. An in‐depth dissection of these metabolic mechanisms to regulate macrophage polarization direction offers substantial theoretical and practical benefits for RA’s targeted therapy.

#### 3.3.3. Epigenetic and Epitranscriptomic Regulation

Beyond the key signaling pathways and energy metabolism pathways mentioned above, epigenetics profoundly influences macrophage function. Current studies have identified that miR‐155 and miR‐221‐3p can promote M1 polarization in RA [46, 59], while circRNA_17725 and circRNA_0066715 can facilitate M2 polarization [60, 61]. N^6^‐methyladenosine (m^6^A) modification is a critical epitranscriptomic modification. Recent studies have revealed that the methyltransferase‐like 3 facilitates the maturation of pri‐miR‐221 by mediating m^6^A modification, thus affecting the polarization balance of RA macrophages, with its expression level correlates positively with how active the disease is [62]. In vitro experiments with cells have revealed that, under inflammatory conditions, Wilms tumor 1‐associated protein expressed in RA FLS can increase m^6^A methylation of the circular RNA circ‐CBLB in exosomes, leading to its degradation and, consequently, encouraging macrophages to shift towards the pro‐inflammatory M1 type [63]. This holds potential value in balancing M1/M2 polarization for RA therapy.

## 4. Targeted Modulation Strategies

To adjust the imbalance in M1/M2 macrophage polarization in RA, researchers are actively exploring targeted therapeutic strategies that inhibit M1 macrophage activity while encouraging a shift towards the anti‐inflammatory M2 type, thereby restoring intra‐articular immune homeostasis and alleviating inflammation and tissue damage.

### 4.1. Novel Intelligent Nano‐Drug Delivery Systems

In the RA synovium, M1 macrophages are the main type, secreting TNF‐α, IL‐1β, and ROS, which play a role in persistent inflammation and bone destruction. Transforming M1 macrophages into M2 macrophages is crucial for reestablishing the immune balance in the joints. Nano‐drug delivery systems can intervene in intracellular signaling by delivering functional molecules to correct the M1/M2 polarization imbalance and offer advantages such as high targeting specificity, enhanced bioavailability, and low systemic toxicity, thereby emerging as a research hotspot in RA therapy.

#### 4.1.1. Microenvironment‐Responsive Systems

The intra‐articular microenvironment in RA patients is characterized by hypoxia, elevated ROS levels, low pH, and elevated MMP levels. Adjusting these conditions can help macrophages switch from the M1 to the M2 phenotype at the location of inflammation. Researchers have accordingly designed various microenvironment‐responsive nanosystems. For instance, Yang et al. [64] constructed a nanomaterial (PCSN@MTX) assembled from molybdenum‐based polyoxometalate, thiolated β‐cyclodextrin, and MTX. This material aggregates in the acidic joint microenvironment and, enhanced by ultrasound, efficiently scavenges ROS, generates oxygen, and downregulates HIF‐1α, thereby reprogramming M1 macrophages into M2 macrophages. Kang et al. [65] employed mesenchymal stem cell (MSC)‐derived apoptotic vesicles that encapsulated dexamethasone (Dex). The surface of these vesicles was further modified with low‐molecular‐weight heparin (LMWH) through a ROS ‐responsive thioketal (TK) linker, thereby generating a functional complex denoted as D@ApoEVFasL∩L. When injected intravenously, the vesicles are directed to and gather in RA joints because of their MSC origin and their ability to home in on inflammation, along with LMWH binding to P‐selectin on inflamed blood vessel cells. LMWH blocks neutrophil recruitment by competitively binding P‐selectin. In a high‐ROS environment, the TK linker breaks, leading to LMWH detachment and exposing FasL on the vesicles. Within the joint, the exposed FasL connects to Fas receptors on activated neutrophils, leading to their apoptosis via the Fas/FasL signaling pathway, which triggers macrophage repolarization. Simultaneously, the released Dex effectively scavenges excess ROS. In vivo and in vitro experiments confirmed that this strategy synergistically blocks neutrophil infiltration, induces their apoptosis, promotes M2 macrophage polarization, and scavenges ROS, thereby triggering an immune cascade, restoring joint immune homeostasis, and significantly ameliorating RA symptoms. To improve administration compliance, Xia et al. [66] integrated cerium/manganese oxide nanoparticles (BSA@NPs‐MTX) with an ROS‐scavenging function into microneedle patches. Through transdermal delivery, these systems locally scavenge ROS, ameliorate hypoxia, and inhibit inflammatory pathways such as NF‐κB, synergistically promoting M2 macrophage polarization. Jia et al. [67] constructed a pH‐responsive, CD44/folate receptor dual‐targeted nano‐delivery system (RBA‐NPs) for delivering roburic acid to treat RA. In an RA rat model, RBA‐NPs effectively targeted inflamed joints, significantly improving the symptoms, reducing inflammatory cytokine levels, and promoting bone repair. Research on mechanisms showed that RBA‐NPs reduce Embden–Meyerhof–Parnas pathway levels in M1 macrophages by blocking the ERK1/2/HIF‐1α/GLUT1 pathway, thus encouraging their transformation into the M2 phenotype, uncovering a new mechanism for regulating macrophage polarization through metabolic reprogramming. Leveraging the characteristic overexpression of MMPs in the RA joint microenvironment, Zhou et al. [68] developed a nano‐therapeutic system named 2‐APB@DGP‐MM. The system employs nanoparticles coated with macrophage membranes to specifically deliver 2‐aminoethyl diphenylborinate (2‐APB). Its core mechanism involves a peptide segment in the nanoparticle carrier that is specifically cleaved by MMP‐2, which is highly expressed in the RA inflammatory microenvironment. Upon reaching the inflamed joint, MMP‐2 cleaves this peptide, triggering the on‐demand release of 2‐APB. The released 2‐APB acts on joint macrophages, reprogramming them from M1 to M2 phenotype, thereby reducing inflammation at its source and aiding in tissue healing. Studies performed in vitro and in vivo showed the system’s outstanding biocompatibility, enhanced cellular absorption, and its ability to significantly reduce synovial inflammation and cartilage damage in a collagen‐induced arthritis (CIA) mouse model. These strategies provide new directions for overcoming the limitations of conventional therapies (Table 1).

**Table 1:** Nanodrug delivery systems for targeted regulation of macrophage polarization.

Category	Nanoparticle	Main preparation technique	Administration route	Advantages	Ref
Microenvironment‐responsive systems	PCSN@MTX	Self‐assembly	Intravenous injection	Responsive to pH, increasing concentration in acidic joints; ultrasound boosts catalytic activity; ultrasound initiates drug release as needed; combined modulation of the immune microenvironment; excellent biocompatibility and stability	[64]
	D@ApoEVFasL∩L	Electroporation	Intravenous injection	ROS‐responsive; multitarget regulation: blocks neutrophil recruitment, induces their apoptosis, promotes M2 polarization, and scavenges excess ROS to rebuild RA microenvironment; MSC‐derived vesicles and LMWH enable targeted delivery to inflamed joints with good biocompatibility	[65]
	BSA@NPs‐MTX	Modified‐SESD	Transdermal microneedle patch	Hypoxia‐responsive; antioxidant and catalase‐like; eliminates ROS to promote M1‐to‐M2 repolarization; MTX synergy; high bioavailability, low toxicity	[66]
	RBA‐NPs	Self‐assembly	Intravenous injection	pH‐responsive; CD44/folate receptor dual targeting; blocks the ERK/HIF‐1α/GLUT1 pathway to inhibit glycolysis, driving M1‐to‐M2 repolarization	[67]
	2‐APB@DGP‐MM	Chemical conjugation and physical self‐assembly	Intra‐articular injection	Drug release responsive to MMP; active targeting through macrophage membrane coating; effective control of macrophage polarization; minimal toxicity, excellent biosafety, high cellular uptake, and extended retention	[68]
Biomimetic nano‐delivery systems	HA@RFM@GP@SIN NPs	Nanoprecipitation	Intravenous injection	Hybrid RBC/FLS membrane coating confers immune evasion and homing targeting; dual anti‐inflammatory and anti‐proliferative synergy; multitargeting, long circulation, excellent biocompatibility and safety	[69]
	AE@SiO2‐MTX	Physical adsorption and chemical crosslinking	Intravenous injection	Adipose‐derived stem cell exosome biomimetic coating; offers high efficiency in targeting, rapid uptake by cells, continuous drug release, and strong biocompatibility	[70]
	MCB@MMs	Modified‐SESD	Intravenous injection	Macrophage membrane biomimetic coating confers inflammatory targeting and immune evasion; self‐oxygenation alleviates hypoxia; good biosafety	[71]
	Col/Cs@ECM	Chemical conjugation	Intra‐articular injection	ECM shell enables active targeting and immune priming; intracellular targeted release; efficient regulation of macrophage polarization; excellent biocompatibility and safety	[72]
Multifunctional integrated nanoplatforms	RP/HP@Mn/L	Chemical conjugation	Intravenous injection	Dual targeting of FLS and macrophages; combines mild photothermal therapy, chemotherapy, ROS scavenging, and O_2_ production for multi‐mechanism synergistic remodeling of synovial microenvironment; nanocapsule structure enhances inflamed tissue penetration	[73]
	Rapa‐FMn@PMS	Physical adsorption and chemical crosslinking	Intra‐articular injection	The self‐propelled movement of nanomotors improves their penetration and retention in joint tissues, while responsive drug release allows for delivery as needed	[74]
	FA@4BC NPs	Nanoprecipitation	Intravenous injection	Combines fluorescence imaging with photodynamic immunomodulatory therapy for active targeting and real‐time assessment of treatment effectiveness	[75]

#### 4.1.2. Biomimetic Nano‐Delivery Systems

Biomimetic systems endow nanocarriers with excellent biocompatibility, targeting ability, and immune evasion by mimicking natural biological structures. Key biomimetic structures include cell membranes, exosomes, proteins, and the extracellular matrix (ECM). Cell membrane camouflage systems utilize membrane structures from source cells to cloak nanoparticles, thereby conferring them with inflammatory targeting and long‐circulating properties. Lin et al. [69] constructed HA@RFM@GP@SIN NPs (red blood cell‐fibroblast‐like synoviocyte hybrid membrane‐coated nanoparticles), which precisely delivered drugs to joint lesions, drove macrophage repolarization, scavenged ROS, and inhibited synovial hyperplasia by intervening in the PI3K/Akt pathway, achieving dual anti‐inflammatory/anti‐proliferative effects. Exosomes, as endogenous intercellular communication carriers, serve as ideal delivery platforms with an inherent targeting potential. Zhu et al. [70] encapsulated AE@SiO_2_‐MTX (MTX‐loaded nanoparticles integrated into adipose‐derived stem cell‐derived exosomes). Upon intravenous injection, these efficiently targeted inflamed joints, induced M2 macrophage polarization, and inhibited synovial invasion. Using proteins such as albumin as dynamic templates to guide nanoscale assembly enables the construction of structurally stable biomimetic carriers. Such systems can also incorporate targeting coatings for precise drug delivery and microenvironment remodeling. Lv et al. [71] utilized bovine serum albumin as a dynamic template, combining MCB@MMs (macrophage membrane‐coated cerium‐based nanoparticles) with MTX to achieve targeted delivery, ROS scavenging, and in situ oxygen supply, encouraging macrophages to transition from the M1 phenotype to the M2 phenotype. This approach overcomes the limitations of conventional synthetic disease‐modifying antirheumatic drugs (DMARDs) and single antioxidants, offering an innovative therapeutic strategy with translational potential. The ECM, as a biomimetic structure, can target and regulate macrophage phenotypes. Duan et al. [72] developed a novel Dex‐loaded Col/Cs@ECM (collagen/chitosan‐derived ECM) core‐shell microsphere system. The ECM shell targets macrophages and initiates immunomodulation, while the Col/Cs core controls Dex release in the acidic intracellular environment, synergistically repolarizing M1 macrophages to M2. In vitro and in vivo RA models have verified its superior anti‐inflammatory effects and high safety and that it represents a new therapeutic strategy for RA with clinical potential (Table 1).

#### 4.1.3. Multifunctional Integrated Nanoplatforms

The complex pathogenesis of RA involves multiple factors and pathways. Multifunctional integrated nanoplatforms combine active targeting, microenvironment‐responsive drug release, and synergistic therapy, aligning well with the multifactorial nature of RA and representing a current hotspot in nanotechnology research. Wu et al. [73] created a versatile nanocapsule called RP/HP@Mn/L, which features a hollow polydopamine carrier infused with manganese ions, containing rapamycin (Rap) and paeoniflorin (Pae), and surface‐modified with LMWH. Rap acts as an upstream core immunomodulator, inhibiting aberrant FLS proliferation and inducing M2 macrophage polarization. Pae provides extensive and gentle anti‐inflammatory and immune‐modulating effects, safeguarding cartilage and preventing bone damage. Mild photothermal therapy not only directly induces apoptosis of inflammatory cells but also mimics physiological hyperthermia, activating pathways like heat shock proteins to create a microenvironment conducive to tissue repair. The trio of components works together to influence various points throughout the pathological process, from immune system imbalance to tissue injury, effectively remodeling the synovial microenvironment. While inhibiting synovitis, this approach also promotes osteoblast differentiation. It achieves the dual therapeutic aims of alleviating joint inflammation and facilitating bone‐cartilage repair by controlling key pathways, such as PI3K‐AKT and MAPK, to inhibit osteoclast activity. Ma et al. [74] designed a composite nanomotor system (Rapa‐FMn@PMS) based on polydopamine‐hybridized mesoporous silica (PMS) nanoparticles as the core carrier. Manganese dioxide (MnO_2_) nanozymes were grown in situ on the surface, followed by folic acid (FA) modification and final physical adsorption of Rap. This device utilizes H_2_O_2_ enriched at the inflamed joint as fuel; MnO_2_ catalyzes its decomposition to produce O_2_, ameliorating hypoxia and propelling the nanomotor’s autonomous movement, enhancing its diffusion and tissue penetration within the joint. The catechol groups in polydopamine confer cartilage adhesion properties, prolonging the retention time at the inflamed site. In the acidic, high‐ROS microenvironment of RA joints, the nanomotor degrades, releasing Rap, SiO_4_^4−^, and Mn^2+^. SiO_4_^4−^ inhibits osteoclastogenesis, while Mn^2+^ and the released Rap promote cartilage regeneration. Additionally, released Rap inhibits the mTOR pathway, activates autophagy in macrophages, eliminates dysfunctional mitochondria, reduces ROS production at its source, and polarizes M1 macrophages to M2. In vitro and in vivo studies confirmed the nanomotor system’s excellent targeted delivery, antioxidant, anti‐inflammatory, and synergistic therapeutic effects for osteochondral repair. Furthermore, Wang et al. [75] developed a folate‐functionalized aggregation‐induced emission (AIE)‐active nanoplatform (FA@4BC NPs). Besides precise targeting, this platform enables fluorescence imaging of activated macrophages in RA by identifying the folate receptor‐β. When laser irradiates FA@4BC NPs, it generates singlet oxygen (^1^O_2_), which efficiently changes macrophage polarization from M1 to M2. In a CIA mouse model, FA@4BC NPs were injected into the joint, and subsequent in vivo imaging revealed that the nanoparticles specifically accumulated in the inflamed joints. Immunohistochemistry revealed significantly decreased expression of the M1 marker inducible nitric oxide synthase (iNOS) and increased expression of the M2 marker CD206, offering a novel multifunctional approach for precision theranostics in RA (Table 1).

Overall, current nano‐delivery strategies targeting macrophage polarization regulation are advancing towards smarter, more biomimetic, and more integrated systems. These strategies not only focus on directly modulating immune cell phenotypes but also deeply integrate the sensing and remodeling of the pathological RA microenvironment. By synergizing multiple therapeutic mechanisms, they offer a promising roadmap for developing highly effective, low‐toxicity novel RA therapies. Despite the promise, several challenges remain: (i) most studies used CIA mouse models with acute inflammation, while chronic RA models with established erosion are rarely tested; (ii) nanomaterials containing heavy metals such as MnO_2_ and cerium still require systematic evaluation of long‐term biosafety and in vivo distribution; and (iii) intra‐articular injection, though effective, is invasive; systemic administration needs to overcome first‐pass clearance and off‐target effects.

### 4.2. Targeted Regulation of Macrophage Polarization by TCM

Rooted in holistic views and principles of syndrome differentiation, TCM demonstrates advantages in regulating immune homeostasis in RA through multicomponent and multitarget synergy. Various studies have demonstrated that TCM can ease RA by adjusting the imbalance between M1 and M2 macrophages.

#### 4.2.1. TCM Monomers

Extensive research indicates that various monomeric components can effectively adjust the M1/M2 polarization balance through the regulation of critical signaling pathways. A variety of components control inflammation by inhibiting central pro‐inflammatory pathways. For example, triptolide can simultaneously inhibit the NF‐κB, PI3K/AKT, and p38 MAPK pathways, driving macrophages toward the M2 phenotype [76]. Koumine specifically inhibits M1 polarization by suppressing the PI3K/AKT pathway [77]. Suberosin promotes M1 to M2 repolarization by targeting the JAK1/STAT3 signaling pathway and activating the STAT6 pathway [78]. Narirutin antagonizes M1 polarization by inhibiting NOD‐like receptor family pyrin domain‐containing 3 (NLRP3) inflammasome activation while simultaneously suppressing osteoclastogenesis, thereby exerting dual protective effects [79]. Some components indirectly regulate the M1/M2 polarization balance by activating endogenous protective pathways and ameliorating the oxidative stress microenvironment. A derivative of sinomenine enhances M2 conversion by allosterically activating heme oxygenase‐1 (HO‐1), providing anti‐inflammatory, antioxidant, and bone‐protective benefits [80]. 4‐Methylcatechol synergistically regulates the nuclear factor erythroid‐2‐related factor 2 (Nrf2)/HO‐1 and NF‐κB/NLRP3 axes, promoting M2 polarization and reducing pyroptosis [81]. Additionally, curcumin has been found to affect multiple pathways, including NF‐κB and STAT, inhibiting M1 polarization and inducing M2 polarization [82], while the aqueous extract of *Saposhnikovia divaricata* (Turcz.) Schischk. (SADS) can simultaneously target TNF‐α and RAGE signaling, modulating the cytokine profile and restoring the M1/M2 balance [83] (Table 2).

**Table 2:** Research progress on natural products and active ingredients targeting macrophage M1/M2 polarization in the treatment of RA.

Active ingredient	Source/origin	Animal/cellmodel	Effect indicators and pathways	Macrophage polarization effect	Ref
Triptolide	*Tripterygium wilfordii* Hook.f.	AA rats	PI3K/Akt↓, NF‐κB↓, MAPK↓, IL‐10↑, IL‐1β↓, IL‐6↓, CXCL8↓, TNF‐α↓, VEGF‐A↓	M1↓, M2↑	[76]
Koumine	*Gelsemium elegans* (Gardner and Champ.) Benth.	AIA rats CIA mouse RAW264.7cells	PI3K/AKT↓, TNF‐α↓, IL‐6↓, IL‐1β↓, IL‐10↑	M1↓	[77]
Suberosin	*Plumbago zeylanica* L.	CIAmouse, RA‐FLS, BMDM	JAK1/STAT6↑, JAK1/STAT3↓, IL‐10↑, TGF‐β↑, TNF‐α↓, IL‐1β↓, IL‐6↓, MMP‐1, 3, 9, 13↓	M1↓, M2↑	[78]
Narirutin	Citrus medicinal materials	CIA mouse, RAW264.7cells, THP‐1cells, BMDM	NF‐κB↓, MAPK↓, NLRs↓, TNF‐α↓, IL‐1β↓, IL‐6↓, IL‐10↑, CD86↓, CD206↑, NLRP3↓, Caspase‐1↓, GSDMD↓, NFATc1↓	M1↓, M2↑	[79]
Sinomenine	*Sinomenium acutum* (Thunb.) Rehd. et Wils.	CIA mouse	Nrf2/HO‐1↑, NF‐κB↓, iNOS↓, COX‐2↓, CXCL10, NO↓, IL‐1β↓, IL‐6↓, TNF‐α↓, CCL2↓, CD206↑, Arg‐1↑, IL‐10↑	M1↓, M2↑	[80]
4‐Methylcatechol	Converted from quercetin (widely present in onions, apples, and various fruits and vegetables)	CIA mouse	Nrf2/HO‐1↑, NF‐κB↓, IL‐10↑, TNF‐α↓, IL‐6↓, IL‐1β↓, NLRP3↓, caspase‐1↓, GSDMD‐NT↓, iNOS↓, ARG‐1↑, ROS↓	M1↓, M2↑	[81]
Curcumin	*Curcuma longa* L.	AIA rats	NF‐κB↓, ANA↓, IL‐1β↓, IL‐8↓, CD68↓	M1↓	[82]
Aqueous extracts of SADS	SADS	(IL1RA^−^/^−^) mouse, RAW264.7 cells	TNF‐α↓, IL‐6↓, RAGE↓, IL‐10↑	M1↓, M2↑	[83]

*Note:* “↓” represents “inhibition” and ”↑” represents “promotion.”

#### 4.2.2. TCM Formulas

TCM formulas, with their complex compositions, exert effects through multitarget, multipathway mechanisms, making them a current research hotspot. The following sections elaborate on the mechanisms and research progress of various classic TCM formulas and empirical prescriptions in regulating macrophage polarization for RA treatment (Table 3).

**Table 3:** Research progress on traditional Chinese medicine compounds in the treatment of rheumatoid arthritis by targeting macrophage M1/M2 polarization.

Traditional Chinese medicine compound	Composition	Experimental model	Mechanism of action	Effect on macrophage polarization	Ref
EMS	*Atractylodes l ancea* (Thunb.) DC., *Phellodendron amurense* Rupr.	RAW264.7 cells	miRNA‐33/NLRP3↓, TNF‐α↓, IL‐18↓, Arg‐1↑, TGF‐β↑, IL‐10↑, iNOS↓, IL‐1β↓	M1↓, M2↑	[27]
SMP	*Atractylodes l ancea* (Thunb.) DC., *Phellodendron amurense* Rupr., *Achyranthes bidentata* Blume.	CIA mouse	TNF‐α↓, PGE2↓, IL‐1β↓, iNOS↓, Arg‐1↑, AA↓, 20‐carboxy‐LTB4↓, 12‐Keto‐LTB4↓, 11,12‐EET↑, DHA↑, DPA↑, DGLA↑, PPARγ↑	M1↓	[84]
WTD	*Aconitum carmichaeli* Debeaux, *Astragalus mongholicus* Bunge, *Glycyrrhiza uralensis* Fisch., *Ephedra sinica* Stapf, *Paeonia lactiflora* Pall.	RAW264.7cells, CIA mouse	NF‐κB↓, TNF‐α↓, IL‐6↓, IL‐1β↓, MCP‐1↓, MMP3↓, PPARγ↑	M1↓, M2↑	[85]
FHLJT	*Sinomenium acutum* (Thunb.) Rehd. et Wils., *Aconitum carmichaelii* Debeaux, *Paeonia lactiflora* Pall., *Reynoutria japonica* Houtt., *Astragalus mongholicus* Bunge, *Rehmannia glutinosa* Steud.	CIA mouse, RAW264.7 cells	PI3K‐Akt↓, AMPK↑, IL‐1β↓, TNF‐α↓, IL‐6↓, IL‐10↑, CP↓, PADI2↓, PADI4↓, IFI30↓, CD74↓, CD206↑, TCR↓, BCR↓, IL‐17A↓, LYN↓, p‐LYN↓, RAC↓, p‐RAC↓, VEGF↓, VEGFR2↓, p‐VEGFR2↓, ANG‐1↓, TIE2↓, MMP3↓, TRAP↓, NFATc1↓, c‐Fos↓, CTSK↓, MMP9↓	M1↓, M2↑	[86]
WGL	*Rhododendron anthropogonoides* Maxim., *Juniperus angosturana* R.P.Adams, *Ephedra sinica* Stapf, *Myricaria platyphylla* Maxim., *Artemisia sieversiana* Ehrh. ex Willd.	BMDM、CIA mouse	JAK2/STAT3↓, NF‐κB↓, TNF‐α↓, IL‐6↓, CD86↓, CD163↑, Arg↑	M1 → M2	[87]
JWJGC	*Cibotium barometz* (L.) J.Sm., *Ptyas dhumnades* Cantor, *Fissistigma polyanthum* (Hook.f. & Thomson) Merr., *Panax notoginseng* (Burk.) F. H. Chen., *Curcuma longa* L., *Sinomenium acutum* (Thunb.) Rehd. et Wils., etc.	CIA mouse	SLC7A11/GSH/GPX4↑, ROS↓, MDA↓, SOD↑, TNF‐α↓, IL‐1β↓, IL‐6↓, TGF‐β1↑, IL‐10↑, ACSL4↓, FTH1↑, NLRP3↓, caspase‐1↓	M1↓, M2↑	[58]

*Note:* “↓” represents “inhibition;” ”↑” represents “promotion;” “M1 → M2” represents “M1‐to‐M2 repolarization.”

Er miao San (EMS), originating from Danxi Xinfa, consists of equal proportions of *Atractylodes l ancea* (Thunb.) DC., *Phellodendron amurense* Rupr. It helps to clear heat and dry dampness, commonly used for treating syndromes where damp‐heat descends. In modern medicine, it is frequently used to address inflammatory conditions such as RA. Liu et al. [27] discovered that EMS modulates macrophage polarization by affecting the miRNA‐33/NLRP3 signaling axis, which results in reduced secretion of inflammatory factors IL‐1β, TNF‐α, and IL‐18.

Sanmiao pill (SMP), recorded in Yixue Zhengzhuan, add *Achyranthes bidentata* Blume to EMS to enhance the downward direction of the formula’s action. It exhibits effects of clearing heat, drying dampness, reducing swelling, and alleviating pain and is clinically used for conditions like foot and knee joint swelling and pain due to damp‐heat pouring downward. Jiang et al. [84] found that SMP inhibits M1 macrophage polarization, reduces synovial inflammation, and ameliorates RA by activating peroxisome proliferator‐activated receptor γ (PPARγ) and promoting its nuclear translocation, thereby inhibiting the HILPDA/DGAT1 pathway, reducing lipid droplet accumulation, and decreasing arachidonic acid storage in lipid droplets and prostaglandin *E*_2_ synthesis.

Wutou decoction (WTD), originating from Shanghan Zabing Lun, comprises *Aconitum carmichaeli* Debeaux, *Astragalus mongholicus* Bunge, *Glycyrrhiza uralensis* Fisch., *Ephedra sinica* Stapf, and *Paeonia lactiflora* Pall. It helps to warm the meridians, dispel cold, eliminate dampness, and alleviate blockages and is commonly used in clinical settings for RA. Lin et al. [85] demonstrated that WTD corrects the M1/M2 macrophage polarization imbalance and restores immune balance and tissue homeostasis through dual regulation of the NF‐κB and PPARγ signaling pathways, eventually reducing synovial inflammation and aiding joint repair in RA. Yao et al. [86] optimized WTD to develop Fuhu Lijie Tang (FHLJT), composed of *Sinomenium acutum* (Thunb.) Rehd. et Wils., *Aconitum carmichaelii* Debeaux, *Paeonia lactiflora* Pall., *Reynoutria japonica* Houtt., *Astragalus mongholicus* Bunge, and *Rehmannia glutinosa* Steud. Transcriptomic analysis identified the PI3K‐Akt and AMPK signaling pathways as core targets. Furthermore, it markedly reduced the abundance of citrullinated proteins in joint tissues of CIA rats, reduced the levels of key antigen‐presenting proteins such as interferon gamma‐inducible protein 30 (IFI30) and CD74, and suppressed the expression of MHC II molecules on M1 macrophages, which ultimately eased synovial inflammation.

Wuweiganlu (WGL) is a classic formula recorded in the Tibetan medical text Four Medical Tantras. It consists of five plateau‐specific herbs: *Rhododendron anthropogonoides* Maxim., *Juniperus angosturana* R.P.Adams, *Ephedra sinica* Stapf, *Myricaria platyphylla* Maxim., and *Artemisia sieversiana* Ehrh. ex Willd. It is commonly used for RA, gout, and various skin diseases. Wen et al. [87] discovered that the aqueous extract of WGL contains various active components such as flavonoids, terpenes, organic oxides, carboxylic acids, and their derivatives. It can shift macrophage polarization from the M1 type to the M2 type, lower the levels of pro‐inflammatory factors TNF‐α and IL‐1β, and enhance the expression of IL‐10 and M2 markers. The molecular mechanism may involve the modulation of inflammatory signaling pathways like JAK2/STAT3 and NF‐κB.

JinWu JianGu capsule (JWJGC), based on the Miao ethnic formula Jinwu Jiangu Fang, is composed of *Cibotium barometz* (L.) J.Sm., *Ptyas dhumnades* Cantor, *Fissistigma polyanthum* (Hook.f. & Thomson) Merr., *Panax notoginseng* (Burk.) F. H. Chen, *Curcuma longa* L., *Sinomenium acutum* (Thunb.) Rehd. et Wils., etc. It is an empirically derived formula used long‐term for RA. Ling et al. [58] found that JWJGC activates the SLC7A11/GSH/GPX4 signaling pathway, reduces ROS accumulation in synovial tissue, decreases malondialdehyde (MDA) activity, increases superoxide dismutase (SOD) activity, and downregulates ferroptosis‐related lipid metabolism pathways. This restores the M1/M2 polarization balance, reduces pro‐inflammatory factor levels (TNF‐α, IL‐1β, and IL‐6), and ameliorates synovial inflammation, providing a new perspective on its modern pharmacological mechanisms.

## 5. Summary and Outlook

The pathological process of RA is highly complex, resulting from the interaction of multiple immune cell types and various molecular regulatory networks, with M1/M2 macrophage polarization imbalance representing a central issue. During the progression of RA, macrophages often exhibit excessive M1 activation and relative M2 deficiency. This imbalance not only maintains ongoing joint inflammation but also significantly contributes to continuous damage and the gradual breakdown of cartilage and bone tissue. A single factor does not determine the direction of macrophage polarization; rather, it is precisely regulated by multiple layers of mechanisms, including various core signaling pathways, energy metabolism patterns, and epigenetic mechanisms. In‐depth investigation and dissection of this regulatory network are essential for building a solid scientific foundation for the development of targeted RA therapies.

Currently, research strategies for RA are shifting from traditional broad‐spectrum anti‐inflammatory towards precise modulation of immune cell phenotypes. Among various strategies for targeted regulation of macrophage polarization, novel smart nanodelivery systems show significant potential. Their design has evolved from initial passive targeting to multifunctional platforms integrating microenvironment responsiveness, active targeting, and biomimetic camouflage, enabling more precise repolarization of macrophages and effective remodeling of the pathological joint microenvironment. Furthermore, TCM holds unique advantages in RA treatment. Leveraging its holistic regulatory characteristics involving multiple components and targets, both active ingredients from single herbs and classic TCM formulas can correct M1/M2 polarization imbalances by intervening in key signaling pathways. This underscores the value of integrating TCM and Western medicine for RA treatment.

Although the M1/M2 polarization paradigm provides a concise framework for understanding the functional heterogeneity of macrophages in RA, intra‐articular macrophages exhibit a high degree of heterogeneity, with phenotypes and functions that extend far beyond the conventional binary classification, and their clinical translation faces multiple obstacles. First, no drugs directly targeting macrophage polarization have yet entered clinical trials for RA. Although existing disease‐modifying antirheumatic drugs and biologics (such as TNF inhibitors and JAK inhibitors) can partially mitigate the inflammatory effects driven by macrophage activation, they are not macrophage‐specific; to date, no therapy has been proven both effective and safe for specifically depleting or reprogramming RA macrophages. Second, the marker systems upon which the M1/M2 classification relies exhibit marked human–mouse species differences. Whereas iNOS/Arg‐1 are widely used as core markers in rodent models, human macrophages do not display iNOS upregulation consistent with that in mice under inflammatory stimulation. Numerous studies of human RA synovial tissue have employed HLA‐DR, CD40, CD80, CD86, and CD276 as M1 markers and CD163, CD206, CD71, and MerTK as M2 markers rather than the iNOS/Arg‐1 system. This asymmetry in marker systems implies that many mechanistic findings regarding polarization regulation derived from mouse models may not be directly extrapolated to human RA synovial macrophages. Third, M1/M2 phenotypic shifts are more likely to represent an accompanying phenomenon following treatment with TNF/IL‐6 inhibitors rather than a primary target amenable to direct pharmacological intervention. Integrating these three points, it becomes evident that relying solely on the M1/M2 binary framework to design targeted therapeutic strategies for RA may overestimate the druggability of polarization reprogramming and underestimate the true complexity of human macrophage heterogeneity; future research should establish multidimensional marker systems based on human synovial tissue and validate the causal relationship between dynamic changes in macrophage subsets and therapeutic response in clinical cohorts.

**Nomenclature**
AIE: Aggregation‐induced emission
ALP: Alkaline phosphatase
C/EBP‐β: CCAAT/enhancer‐binding protein‐β
CIA: Collagen‐induced arthritis
COL1A1: Collagen type I alpha 1 chain
Dex: Dexamethasone
DLL4: Delta‐like ligand 4
DMARDs: Disease‐modifying antirheumatic drugs
ECM: Extracellular matrix
EMS: Er Miao San
FA: Folic acid
FcγRII: Fc gamma receptor II
FHLJT: Fuhu Lijie Tang
FLS: Fibroblast‐like synoviocytes
GPx4/GPX4: Glutathione peroxidase 4
HIF‐1 α: Hypoxia‐inducible factor 1α
HMGB1: High‐mobility group box‐1
HO‐1: Heme oxygenase‐1
IDO: Indoleamine 2,3‐dioxygenase
IFI30: Interferon gamma‐inducible protein 30
IL‐10: Interleukin‐10
IL‐1β: Interleukin‐1β
IL‐6: Interleukin‐6
iNOS: Inducible nitric oxide synthase
JAK/STAT: Janus kinase/signal transducer and activator of transcription
JWJGC: JinWu JianGu capsule
LMWH: Low‐molecular‐weight heparin
LPS: Lipopolysaccharide
M0: Unpolarized resting macrophages
m^6^A: N^6^‐methyladenosine
MAPK: Mitogen‐activated protein kinase
MDA: Malondialdehyde
MDMs: Monocyte‐derived macrophages
MHC II: Major histocompatibility complex class II
MMPs: Matrix metalloproteinases
MnO_2_: Manganese dioxide
MSC: Mesenchymal stem cell
MTX: Methotrexate
NF‐κB: Nuclear factor‐kappa B
NGF: Nerve growth factor
NLRP3: NOD‐like receptor family pyrin domain‐containing 3
Nrf2: Nuclear factor erythroid‐2‐related factor 2
OCN: Osteocalcin
OPG: Osteoprotegerin
OPN: osteopontin
OXPHOS: Oxidative phosphorylation
Pae: Paeoniflorin
PDGF: platelet‐derived growth factor
PI3K/Akt: Phosphatidylinositol 3‐kinase/protein kinase B
PMS: Polydopamine‐hybridized mesoporous silica
PPARγ: Peroxisome proliferator‐activated receptor γ
RA: Rheumatoid arthritis
RANK: Receptor activator of nuclear factor‐κB
RANKL: Receptor activator of nuclear factor‐κB ligand
Rap: Rapamycin
ROS: Reactive oxygen species
RUNX2: Runt‐related transcription factor 2
SADS: *Saposhnikovia divaricata* (Turcz.) Schischk.
SMP: Sanmiao pill
SOCS‐1: Suppressor of cytokine signaling‐1
SOD: Superoxide dismutase
TCA: Tricarboxylic acid cycle
TCM: Traditional Chinese medicine
TGF‐β: Transforming growth factor‐β
TK: Thioketal
TLR4: Toll‐like receptor 4
TNF‐α: Tumor necrosis factor‐α
TRAP: Tartrate‐resistant acid phosphatase
TRMs: Tissue‐resident macrophages
VEGF: Vascular endothelial growth factor
WGL: Wuweiganlu
WTD: Wutou decoction
^1^O_2_: Singlet oxygen.

## Author Contributions

**Feng Hao:** writing – review and editing, supervision, funding acquisition. **Jun Hu:** writing – review and editing, supervision, project administration. **Hui Zuo:** writing – original draft, writing – review and editing. **Jiajia Sang:** conceptualization, writing – original draft, writing – review and editing. **Enqi Fan:** writing – original draft, writing – review and editing, visualization. **Long Zeng:** writing – review and editing.

## Funding

This work was supported by the Program of the National Natural Science Foundation of China (Grant 82274645).

## Disclosure

All authors have read and approved the final manuscript.

## Conflicts of Interest

The authors declare no conflicts of interest.

## Data Availability

No datasets were generated or analyzed in the current study.
